# A Reciprocal Excitatory Reflex Between Extensors Reproduces the Prolongation of Stance Phase in Walking Cats: Analysis on a Robotic Platform

**DOI:** 10.3389/fnbot.2021.636864

**Published:** 2021-04-08

**Authors:** Toyoaki Tanikawa, Yoichi Masuda, Masato Ishikawa

**Affiliations:** Department of Mechanical Engineering, Osaka University, Suita, Japan

**Keywords:** spinal reflex, neurophysiology, bio-inspired robot, quadruped, walking, gait generation, hill-type muscle, autonomous decentralized control

## Abstract

Spinal reflex is essential to the robust locomotion of quadruped animals. To investigate the reflex mechanisms, we developed a quadruped robot platform that emulates the neuromuscular dynamics of animals. The leg is designed to be highly back-drivable, and four Hill-type muscles and neuronal pathways are simulated on each leg using software. By searching for the reflex circuit that contributes to the generation of steady gait in cats through robotic experiments, we found a simple reflex circuit that could produce leg trajectories and a steady gait. In addition, this circuit can reproduce the experimental behavior observed in cats. As a major contribution of this study, we show that the underlying structure of the reflex circuit is the reciprocal coupling between extensor muscles via excitatory neural pathways. In the walking experiments on the robot, a steady gait and experimental behaviors of walking cats emerged from the reflex circuit without any central pattern generators. Furthermore, to take advantage of walking experiments using a neurophysiological robotic platform, we conducted experiments in which a part of the proposed reflex circuit was disconnected for a certain period of time during walking. The results showed that the prolongation of the stance phase caused by the reciprocal excitatory reflex contributed greatly to the generation of a steady gait.

## 1. Introduction

Quadruped animals can immediately respond to various environmental disturbances and achieve steady locomotion. Many experiments have been conducted to reveal the mechanism of motion generation in quadrupeds. Grillner (1975) reported that the central pattern generator (CPG) in the spinal cord generates a rhythmic gait pattern, even when the nerves from the brain and proprioceptors are suspended. However, sensory feedback from receptors contributes significantly to the motion generation of animals. Pearson (2004) demonstrated that sensory feedback through reflex pathways determines the timing of phase transitions in a step cycle and shapes the characteristics of movement patterns, which contributes significantly to extensor activation in walking cats.

Functions of the motor coordination of reflexes in walking quadrupeds have been investigated in animal experiments. In experiments by Whelan et al. (1995), electrical stimulation on the afferent nerves from ankle extensor muscles prolonged the stance phase in walking cats. This result suggests that the unloading of the ankle extensor muscles initiates the stance-to-swing transition. In experiments by McVea et al. (2005), assisting the flexor muscle movement in walking cats during the swing phase accelerated the activation timing of the ankle extensor muscles. This result suggests that the angle of the hip joint initiates the swing-to-stance transition. These studies highlight individual reflex mechanisms in stance and swing phases; however, the entire reflex circuit that generates the walking motion has not yet been identified.

In recent years, the constructivist approach has been employed to investigate the locomotion mechanism of animals by reproducing motor control of animals using robots and computer simulations. Habu et al. (2018) has proposed a neuromuscular model that reproduces the musculoskeletal system and detailed neural pathways of cats. In the walking simulation, they designed a CPG network to generate a trot gait, and when they introduced feedback of ground reaction forces into the CPG, the model changed the gait from trot to walk and gallop. As a simpler model to understand the locomotion mechanism of animals, Maufroy et al. (2010), Aoi et al. (2013), and Owaki and Ishiguro (2017) proposed an oscillator model that adjusts leg phase based on the ground contact information. In a walking experiment, their robots can produce multiple animal-like gait patterns depending on the speed.

Several studies have shown a crucial result in this research field that the leg trajectories and steady gait can be achieved by the interaction between spinal reflexes, body dynamics, and environment, without using oscillator models or complex CPG models. A human musculoskeletal model of Geyer and Herr (2010) produced steady alternating gait using only reflex rules. A cat hind leg model of Ekeberg and Pearson (2005) also showed alternative gait only with the reflex rules, and Rosendo et al. (2014) investigated this idea in real-world experiments using a musculoskeletal robot. However, in these studies, the designers divided the walking motion into multiple phases (ex. stance, liftoff, swing, and touchdown phase) and designed a separate reflex rule for each phase. Therefore, it is not clear how these many reflex rules are integrated in the animal body, i.e., the overall structure of the reflex circuit that produces a steady gait and leg trajectory.

In order to clarify the structure of the reflex circuit that generates the steady locomotion of cats, we explored the reflex circuit using a quadruped robot platform that emulates the neuromuscular dynamics of animals.

As a result, we found a simple reflex circuit that could produce a steady gait and leg trajectories and also reproduce the experimental behavior of cats. The major contribution of this study is clarifying the essential structure of the reflex circuit to produce a steady gait, which is the reciprocal excitatory reflex between hip and knee–ankle extensor muscles. To evaluate the proposed reflex circuit, we conducted walking experiments and reproduced a neurophysiological experiment based on cats on a quadruped robot. In the walking experiments, the quadruped robot did not have a central pattern generator; however, it produced a gait pattern and leg trajectories autonomously. In the reproduction experiment of cats' walking behaviors, the robot reproduces the swing-to-stance transition based on the hip angle in McVea et al. (2005) and a prolongation function of the stance phase, with stimulation on the ankle extensor nerves, as in Whelan et al. (1995). Moreover, utilizing a robot with a reprogrammable reflex law in real time, we conducted an experiment to remove the reciprocal excitatory pathway between the extensors (the prolongation function of the stance phase) during walking. The absence of the reciprocal excitatory pathway reduced the gait stability of the robot. This result suggests that the prolongation function provided by the reciprocal excitatory pathway between the hip and knee–ankle extensors stabilizes the gait pattern.

In section 2, we explain the mechanical design and control system of the quadruped robot platform. Sections 3 and 4 describe muscle and reflex model and their implementation to the robot. Section 5 presents the result of walking experiments, and the gait emergence mechanism and a comparison with a previous study are discussed in section 6. Section 7 summarizes the effect of proposed reflex circuit and addresses about future work.

## 2. Quadruped Robot That Reproduces Muscular Properties and Reflexes

**Figure 1:**
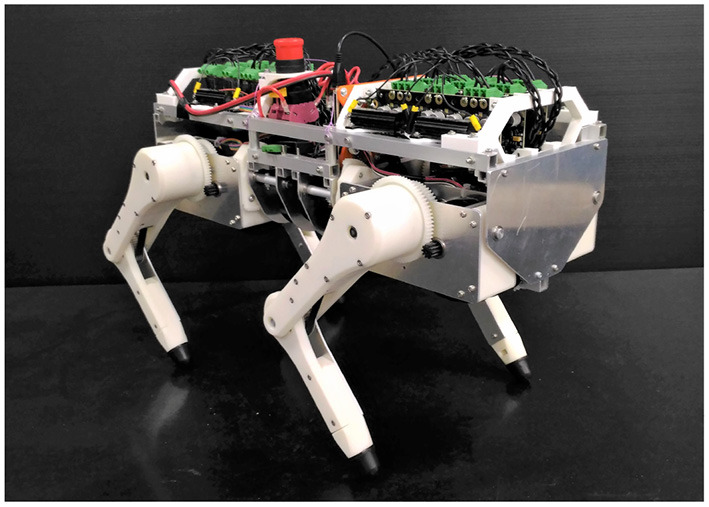
Snapshot of a quadruped robotic platform that can reproduce muscle characteristics and reflexes.

To reproduce and understand the reflex mechanism of animals, we construct a quadruped robotic platform, shown in Figure 1, that can reproduce muscle characteristics and reflexes like those in Zhao et al. (2020). This quadruped robot comprises highly back-drivable legs to reproduce the flexibility of animals and torque-controllable motors that enable the robot to emulate the muscle characteristics virtually.

**Figure 2:**
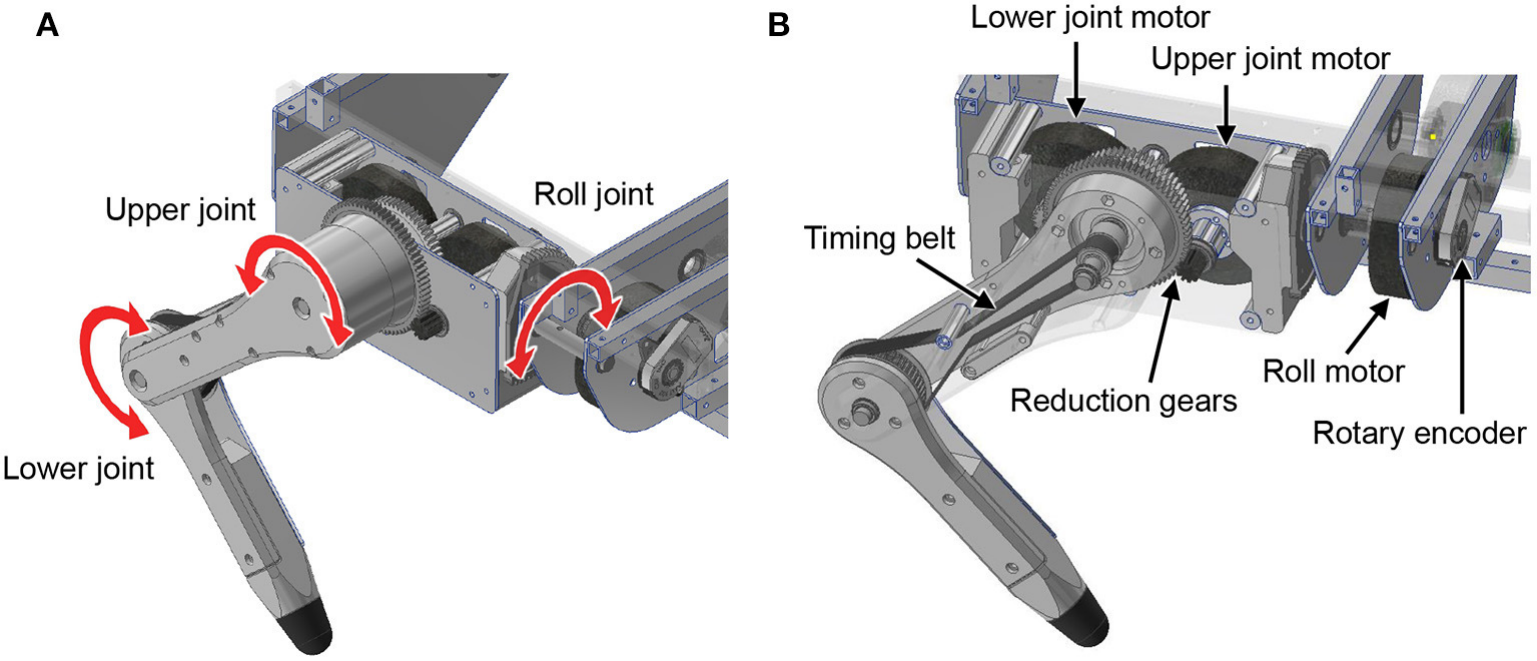
Leg design of the quadruped robot. **(A)** Degree of freedom for the legs. **(B)** Detailed mechanism of the legs.

First, we explain the mechanical design of the quadruped robot platform. As shown in Figure 2, each leg consists of two links, and the legs can move freely in the sagittal plane by driving the upper and lower rotational joints. The leg module can also rotate in the roll direction as adduction and abduction motion; thus, the legs have three degrees of freedom. Each joint is driven by a brushless direct current (BLDC) motor (MN6007 KV160, T-Motor, China). The torque is transmitted by gears and timing belts in the lower rotational joints and by only gears in the other two joints. We embed the motors inside the hip part of the leg module to reduce the moment of leg inertia. The reduction ratio is as low as 1:7 so that all joints achieve high back-drivability, and the measurement of motor current enables torque control. A rotary encoder (ATM102-V, CUI Devices, USA) is installed on the back of each motor to measure the rotation angle. The body length, distance between the hips, link length of the legs, and weight are 470 mm, 289 mm, 152 mm, and 7.6 kg, respectively.

Next, we describe the control system of the quadruped robot. This control system comprises a low-level controller for the BLDC motor and a high-level controller that reproduces the muscle characteristics and reflexes. The motor driver (v3.6 24V, ODrive) provides low-level control for the motor torque with current feedback and measures the angle using the rotary encoder at a frequency of 8 kHz. A microcontroller (Teensy 3.6) provides high-level control. It emulates virtual muscles and reflex circuits based on the angle information received from the motor driver and commands the target torques to reproduce their characteristics to the motor driver. The communication frequency between the controllers is 1 kHz.

## 3. Reproduction of Muscle Properties

To reproduce and understand the reflex mechanisms of animals, we emulate muscles and neural models using a quadruped robot software.

### 3.1. Muscle Model

**Figure 3:**
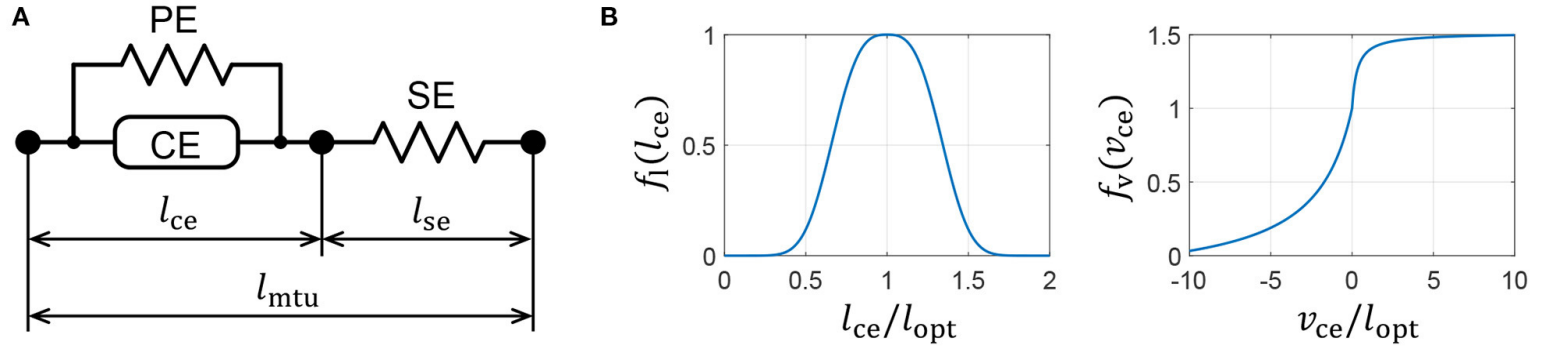
Muscle properties reproduced on a quadruped robot. **(A)** Hill-type muscle model. **(B)** Force–length and force–velocity characteristics of the contractile element (CE).

We introduce a Hill-type muscle model developed by Geyer and Herr (2010), which is shown in Figure 3A. The Hill-type muscle model consists of a contractile element (CE) that exerts tension according to the muscle activation, a parallel-connected elastic element (PE), and a series-connected elastic element (SE). The CE has a length-dependent property, wherein the contraction force is maximized at an optimal length, and a velocity-dependent property. The contraction force becomes relatively small and large during contraction and extension, respectively. The elastic elements, PE and SE, have non-linear elastic properties that produce tension when they exceed a particular length.

Next, we formulate the details of the Hill-type muscle model developed by Geyer and Herr (2010). Using muscle activity *A* ∈ [0, 1], we express the generated CE force, *F*_ce_, as

(1) $$\begin{array}{l} F_{\text{ce}}=AF_{\text{max}}f_{\text{l}}\left(l_{\text{ce}}\right)f_{\text{v}}\left(v_{\text{ce}}\right), \end{array}$$

where *F*_max_ is the maximum isometric force, and *l*_ce_ and *v*_ce_ are the length and velocity of the CE, respectively. The force–length and force–velocity characteristics, *f*_l_(*l*_ce_) and *f*_v_(*v*_ce_), respectively, are represented as

(2) $$\begin{array}{l} f_{\text{l}}\left(l_{\text{ce}}\right)=\exp\left(c|\frac{l_{\text{ce}}-l_{\text{opt}}}{l_{\text{opt}}\omega }{|}^{3}\right) \end{array}$$

(3) $$\begin{array}{l} f_{\text{v}}\left(v_{\text{ce}}\right)=\{\begin{array}{ll} \frac{v_{\text{max}}-v_{\text{ce}}}{v_{\text{max}}+Kv_{\text{ce}}} & v_{\text{ce}}<0\\ N+\left(N-1\right)\frac{v_{\text{max}}+v_{\text{ce}}}{7.56Kv_{\text{ce}}-v_{\text{max}}} & v_{\text{ce}}\geq 0 \end{array}, \end{array}$$

where *l*_opt_ is the length of *f*_l_(*l*_opt_) = 1, and *c*, ω, *v*_max_, *K*, and *N* are parameters that are used to determine the muscle characteristics. The graphs of functions *f*_l_(*l*_ce_) and *f*_v_(*v*_ce_) are presented in Figure 3B. The PE force, *F*_pe_, and SE force, *F*_se_, are expressed as

(4) $$\begin{array}{l} F_{\text{pe}}(l_{\text{ce}})=\{\begin{array}{ll} F_{\text{max}}{\left(\frac{l_{\text{ce}}-l_{\text{opt}}}{l_{\text{opt}}\epsilon_{\text{pe}}}\right)}^{2} & l_{\text{ce}}>l_{\text{opt}}\\ 0 & l_{\text{ce}}\leq l_{\text{opt}} \end{array} \end{array}$$

(5) $$\begin{array}{l} F_{\text{se}}\left(l_{\text{se}}\right)=\{\begin{array}{ll} F_{\text{max}}{\left(\frac{l_{\text{se}}-l_{\text{slack}}}{l_{\text{slack}}\epsilon_{\text{ref}}}\right)}^{2} & l_{\text{se}}>l_{\text{slack}}\\ 0 & l_{\text{se}}\leq l_{\text{slack}} \end{array}, \end{array}$$

where *l*_se_ is the SE length, *l*_slack_ is the rest length of SE, and ϵ_pe_ and ϵ_ref_ are the reference strains of PE and SE, respectively.

### 3.2. Implementation of Muscle Models to the Robot

**Figure 4:**
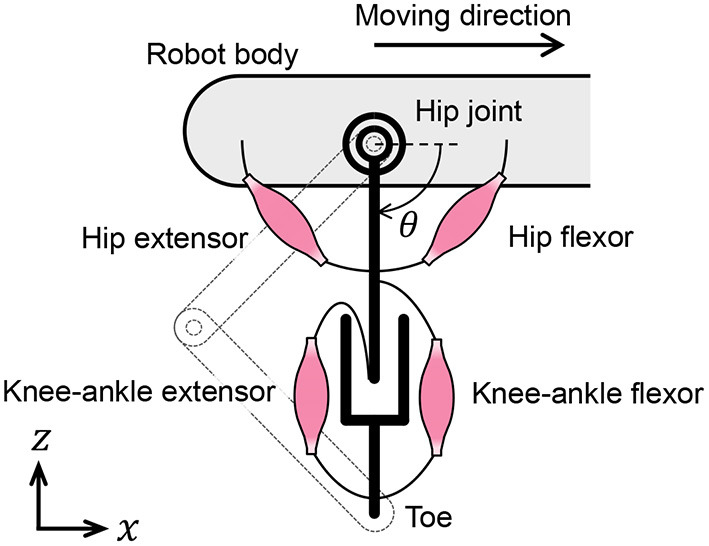
Placement of muscle models on the quadruped robot.

In this subsection, we discuss the muscle placement on the quadruped robot. Although quadruped animals have numerous muscles in their legs, we classify them into radial and angular muscles based on the direction of force generation to improve our understanding of the walking phenomena. According to the muscle classification, the two-link legs of the quadruped robot are considered to possess virtual rotational hip joints and linear joints between the hips and toes, as shown in Figure 4. The extensors and flexors are placed on each virtual joint. The muscles of virtual rotational and linear joints approximately correspond to the hip muscles and knee–ankle muscles in animals, respectively.

We reproduce the muscle characteristics in the quadruped robot using the following method developed by van Soest and Bobbert (1993):

1) Compute muscle length *l*_mtu_ from the measured leg joint angles.
2) Update CE length *l*_ce_. Because the SE is connected in series to the CE and the PE, the muscle force, *F*_mtu_, is expressed as
  (6) $$\begin{array}{l} F_{\text{mtu}}=F_{\text{se}}=F_{\text{ce}}+F_{\text{pe}}. \end{array}$$
  Using Equations (1) and (6), the velocity of the CE is deformed as
  (7) $$\begin{array}{l} v_{\text{ce}}=\frac{dl_{\text{ce}}}{dt}=f_{\text{v}}^{-1}\left[\frac{F_{\text{se}}\left(l_{\text{se}}\right)-F_{\text{pe}}\left(l_{\text{ce}}\right)}{AF_{\text{max}}f_{\text{l}}\left(l_{\text{ce}}\right)}\right], \end{array}$$
  and *l*_ce_ is computable by integrating Equation (7).
3) The muscle force, *F*_mtu_, is obtained by calculating *F*_se_ in Equation (5) using *l*_se_ = *l*_mtu_ − *l*_ce_. The muscle characteristics are reproduced by generating motor torques that correspond to the muscle forces.

Please see Geyer and Herr (2010) for more detail.

## 4. Implementation of the Reflex Circuit

To reproduce and understand the reflex mechanism in animal walking, we construct a reflex circuit that can reproduce the walking functions of cats. In this section, we propose a simple reflex circuit based on the results of previous experiments on walking cats.

### 4.1. Design of the Reflex Pathway

We describe two results from previous experiments on walking cats to construct a reflex pathway for the quadruped robot. In experiments by Whelan et al. (1995), electric stimulation on afferent nerves from ankle extensor muscles prolonged the stance phase. This result suggests that the unloading of ankle extensor muscles initiates the stance-to-swing transition. In experiments by McVea et al. (2005), assisting the flexor muscle movement during the swing phase accelerated the activation timing of the ankle extensor muscles. This suggests that the angle of the hip joint initiates the swing-to-stance transition.

**Figure 5:**
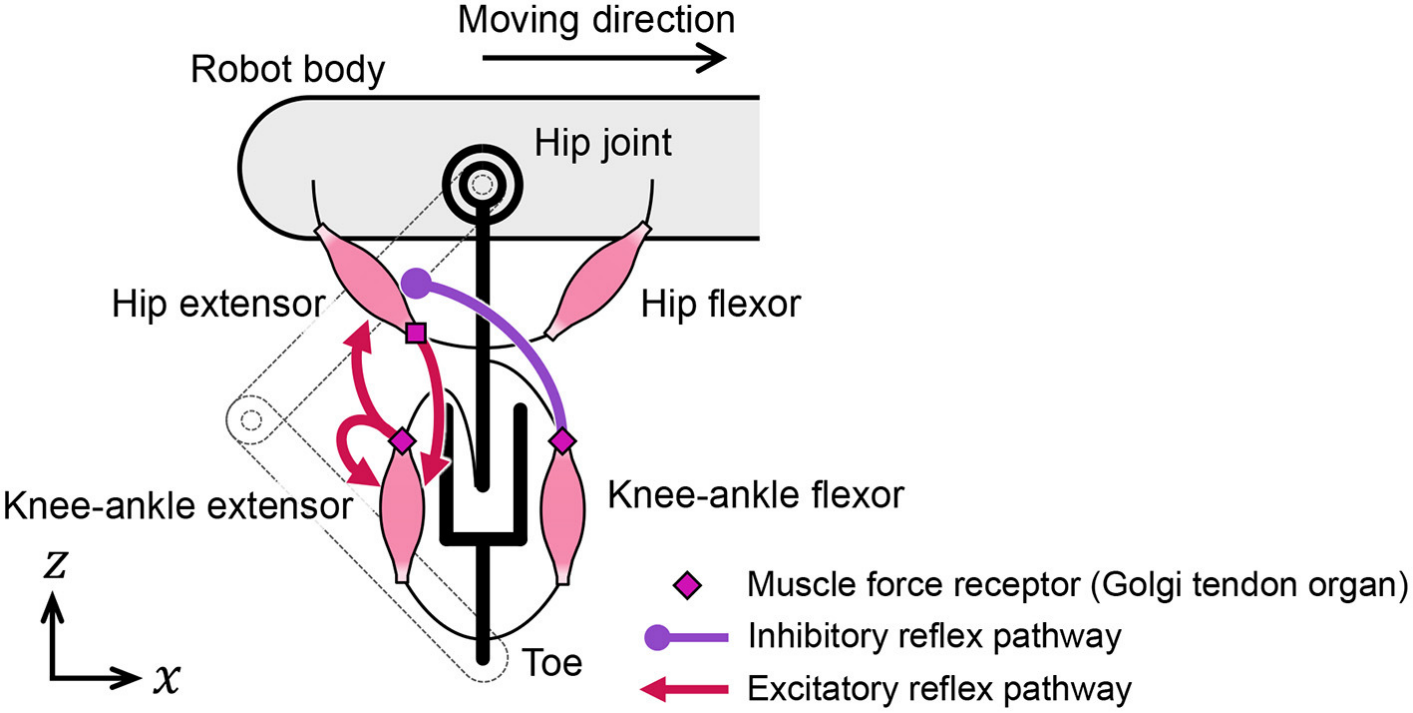
Reflex pathways embedded in the quadruped robot.

We designed the reflex pathways, shown in Figure 5, using these results. From the first experiment, we embedded excitatory reflex pathways from the force receptors of the knee–ankle extensors to the hip and knee–ankle extensor muscles. These pathways provide a function; if the knee–ankle receptors continue to sense the ground reaction force, the muscles continue to extend the knee–ankle and hip joints. From the second experiment, we embedded an excitatory reflex pathway from the force receptors of the hip extensors to the knee–ankle extensor muscles. With this pathway, if the hip extensor muscles are fully stretched and generate tension, the sensory signal initiates the swing-to-stance transition. It is important to note that to suppress excessive excitation of the hip extensor at the end of the swing phase, we embedded an inhibitory reflex pathway from the force receptors of knee–ankle flexors to hip extensors through trial and error.

### 4.2. Modeling of the Reflex Circuit

**Figure 6:**
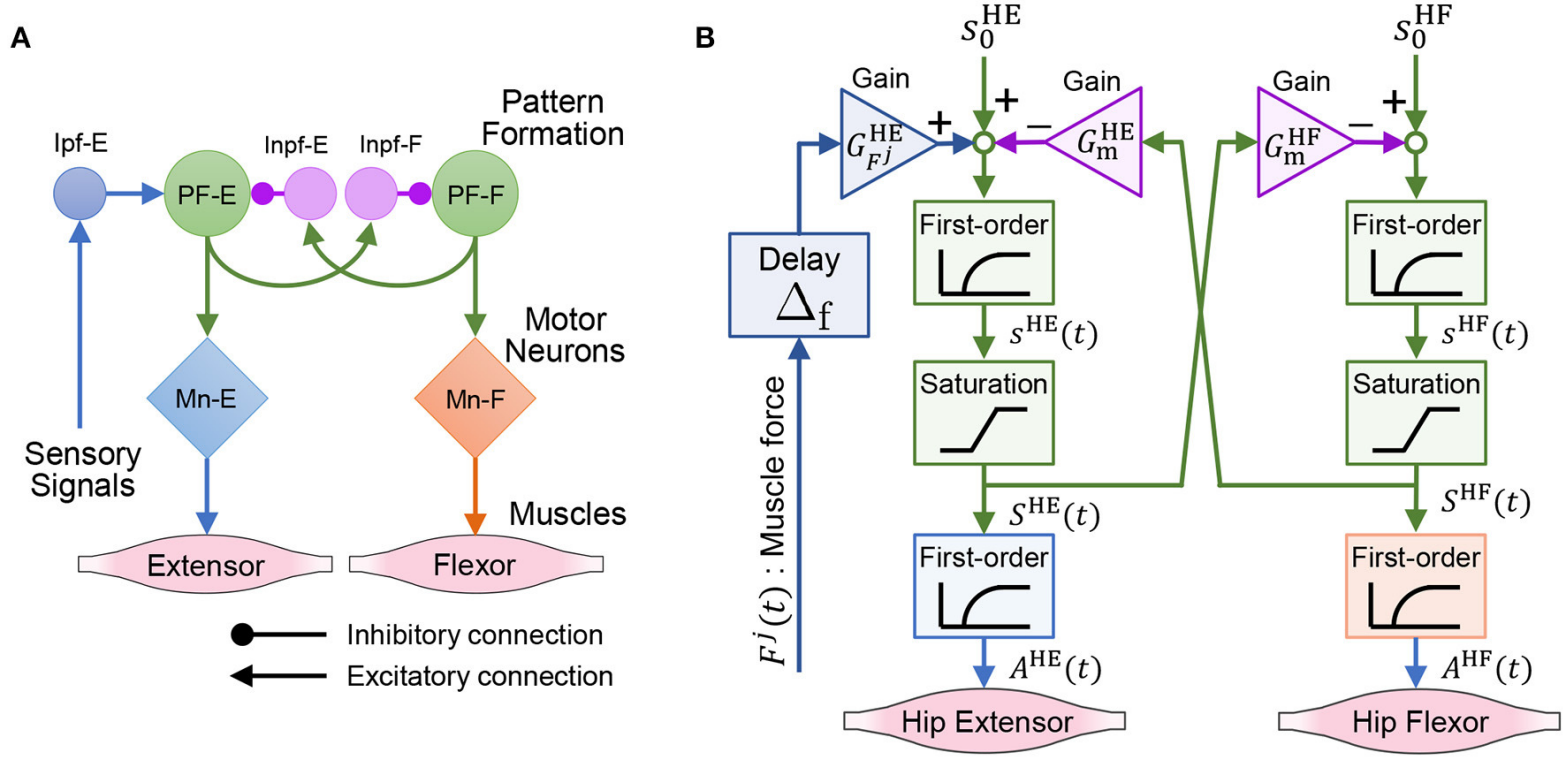
Neural circuit reproduced for the quadruped robot. **(A)** Reflex circuit extracted from the neural circuit model by Rybak et al. (2006). **(B)** Implementation model of the reflex circuit for the hip muscles. The same neural circuit is used for the knee–ankle muscles.

This subsection introduces a model of the spinal reflex that can generate walking motions with the quadruped robot. We implemented a simple reflex circuit, shown in Figure 6A, which is part of the neural circuit model proposed by Rybak et al. (2006).

We formulate a model of the reflex circuit. In the following equations, the superscript set of the leg muscles is denoted as M={HE,HF,KE,KF}, where HE, HF, KE, and KF denote the hip extensors, hip flexors, knee–ankle extensors, and knee–ankle flexors, respectively. For the state of a muscle, i∈M, at time *t*, we denote the muscle activation as *A*^*i*^(*t*), the muscle force scaled by *F*_max_ as *F*^*i*^(*t*), and the excitation of the interneuron as *S*^*i*^(*t*).

The implementation model of the reflex circuit is shown in Figure 6B. The behavior of a motor neuron that determines muscle activity *A*^*i*^(*t*) is expressed by Geyer et al. (2003) as

(8) $$\begin{array}{l} \tau_{\text{mn}}\frac{dA^{i}\left(t\right)}{dt}+A^{i}\left(t\right)=S^{i}\left(t\right), \end{array}$$

where τ_mn_ is a time constant of the excitation-contraction coupling. The excitation of the interneuron, *S*^*i*^(*t*), is given by

(9) $$\begin{array}{l} \tau_{\text{in}}\frac{ds^{i}\left(t\right)}{dt}+s^{i}\left(t\right)=s_{0}^{i}+\underset{j\in M}{\sum }G_{F^{j}}^{i}F^{j}\left(t-\Delta_{\text{f}}\right)-G_{\text{m}}^{i}S^{ī}\left(t\right) \end{array}$$

(10) $$\begin{array}{l} S^{i}\left(t\right)=\{\begin{array}{ll} 0 & s^{i}\left(t\right)<0\\ s^{i}\left(t\right) & 0\leq s^{i}\left(t\right)\leq 1\\ 1 & s^{i}\left(t\right)>1 \end{array}, \end{array}$$

where *s*^*i*^(*t*) is the internal state of the interneuron. The second term on the right-hand side of Equation (9) expresses the reflex pathways that feedback muscle force to the interneurons, and the third term expresses the reciprocal inhibition pathways of the interneurons. In Equation (9), superscript $\bar{i}\in M$ is the antagonist muscle of muscle *i*, and ī represents HF, HE, KF, and KE, when *i* is HE, HF, KE, and KF, respectively. Moreover, $s_{0}^{i}$ is the bias of the neural input, τ_in_ is the time constant of the interneurons, $G_{F^{j}}^{i}$ is the gain of the muscle force feedback, $G_{\text{m}}^{i}$ is the gain of the reciprocal inhibition, and Δ_f_ is the time delay of the signal propagation.

## 5. Walking Experiments Using the Quadruped Robot With Reflex and Muscle Characteristics

To evaluate the proposed reflex circuit based on cat walking behaviors, we conducted walking experiments and reproduced a physiological experiment based on cats on the quadruped robot. In addition, by utilizing a robot with a reprogrammable reflex law in real time, we conducted an experiment to remove the reciprocal excitatory pathway between the extensors (the prolongation function of the stance phase) during walking.

### 5.1. Experimental Conditions

The parameters for the hip muscles were set as *l*_opt_ = π/6 rad, *l*_slack_ = π/6 rad, and *F*_max_ = 5 N·m, and those for the knee–ankle muscles were set as *l*_opt_ = 80 mm, *l*_slack_ = 80 mm, and *F*_max_ = 80 N. Additionally, we positioned the muscles to satisfy *l*_ce_ = 0.9*l*_opt_ for the hip muscles and *l*_ce_ = 0.8*l*_opt_ for the knee–ankle muscles when the toe coordinates with respect to the hip joint are (*x, z*)= (0 m, −0.21 m). Other muscle parameters were obtained from Geyer and Herr (2010).

We applied a virtual spring-damper characteristic to the roll joint of the leg module with a natural angle in the downward direction. The spring constant was set to 100 N·m/rad, and the damping factor was set to 1 N·m·s/rad.

The parameters of the reflex circuit in Equation (9) are presented in Table 1. In Table 1, $G_{F^{\text{KE}}}^{\text{HE}}$, $G_{F^{\text{KF}}}^{\text{HE}}$, $G_{F^{\text{HE}}}^{\text{KE}}$, and $G_{F^{\text{KE}}}^{\text{KE}}$ represent the gains for the excitatory feedback from the knee–ankle extensor to the hip extensor, the inhibitory feedback from the knee–ankle flexor to the hip extensor, the excitatory feedback from the hip extensor to the knee–ankle extensor, and the self-excitatory feedback of the knee–ankle extensor, respectively. The parameters of the reflex circuit were adjusted to generate a walking motion. The delay time of the muscle information, Δ_f_, was set to 15 ms, and the time constants of the motor neurons, τ_mn_, and interneurons, τ_in_, were set to 10 and 5 ms, respectively.

**Table 1:** Values of the reflex circuit parameters in Equation (9).

**i**	**$s_{0}^{i}$**	**$G_{F^{\text{HE}}}^{i}$**	**$G_{F^{\text{HF}}}^{i}$**	**$G_{F^{\text{KE}}}^{i}$**	**$G_{F^{\text{KF}}}^{i}$**	**$G_{\text{M}}^{i}$**
HE	0	0	0	1.2	−1	0.15
HF	1	0	0	0	0	10
KE	−0.1	4	0	1.5	0	0
KF	1	0	0	0	0	10

**Figure 7:**
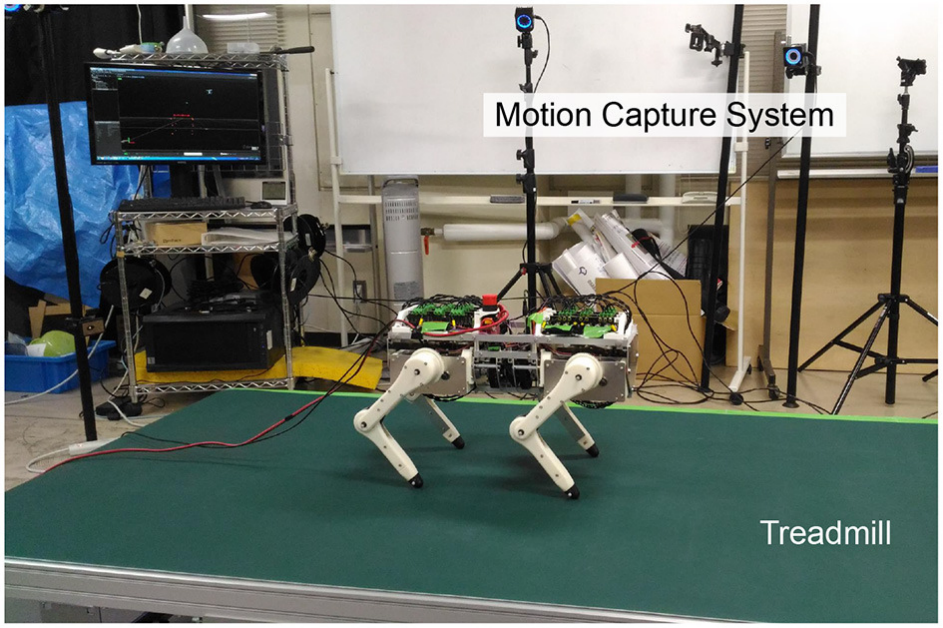
Snapshot of the experimental environment.

Figure 7 depicts the experimental environment. The quadruped robot walked on a treadmill, and a motion capture system measured its movements.

### 5.2. Walking Experiment on a Treadmill

First, we conducted a walking experiment using the proposed reflex circuit^1^. The periodic motion of the legs did not appear in the air because this robot, which has a reflex circuit, generates motion via its interaction with the environment. At the beginning of the walking experiment, we held the robot with each leg at rest and placed it on a treadmill to activate the reflex circuit.

**Figure 8:**
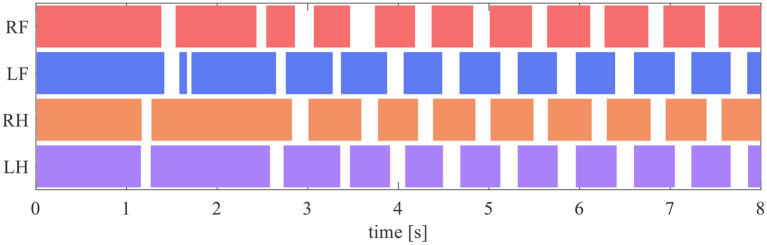
Gait diagram. RF, LF, RH, and LH represent the right–fore leg, left–fore leg, right–hind leg, and left–hind leg, respectively, and the colored regions indicate ground contact.

The quadruped robot generated a steady gait, even though it did not contain a central rhythm generator or pattern generator. Figure 8 presents the gait diagram of the robot. RF, LF, RH, and LH in Figure 8 represent the right–fore leg, left–fore leg, right–hind leg, and left–hind leg, respectively, and the colored regions indicate ground contact. The result reveals that the ground contact timing of each leg is gradually adjusted with time. After 4 s, the timing of the stance phases between RF–RH and LF–LH were the same, and each of them was in antiphase; thus, a pace gate emerged.

**Figure 9:**
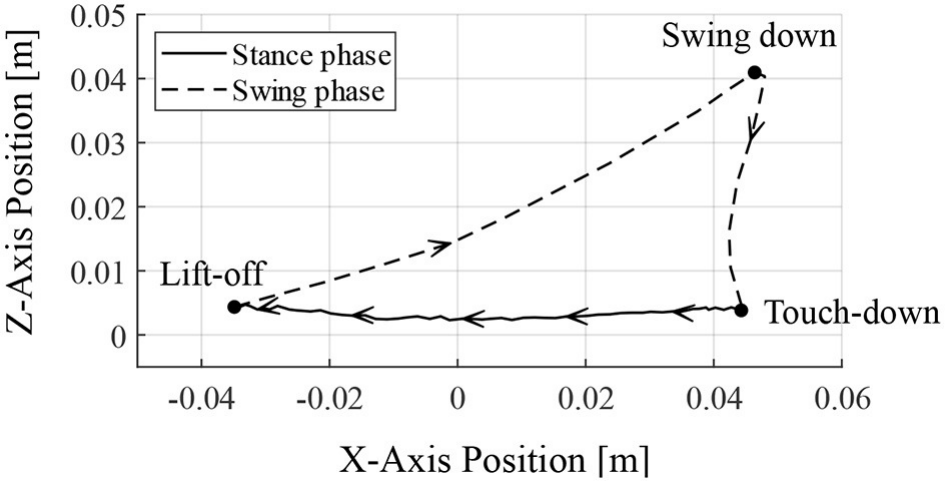
Toe trajectory of the right–hind leg. The foot position is based on the hip joint in the x-axis and the ground in the z-axis. Only one cycle of walking is shown from the time of ground contact at 6.95 s.

Figure 9 depicts the toe position of the RH leg with respect to the hip joint (in the x-axis) and the ground (in the z-axis) for one cycle of walking from 6.95 s, when the leg contacted the ground. The arrows in Figure 9 indicate the direction of the toe movement. The result indicates that the proposed reflex circuit produces a walking trajectory autonomously, without a pre-designed trajectory.

**Figure 10:**
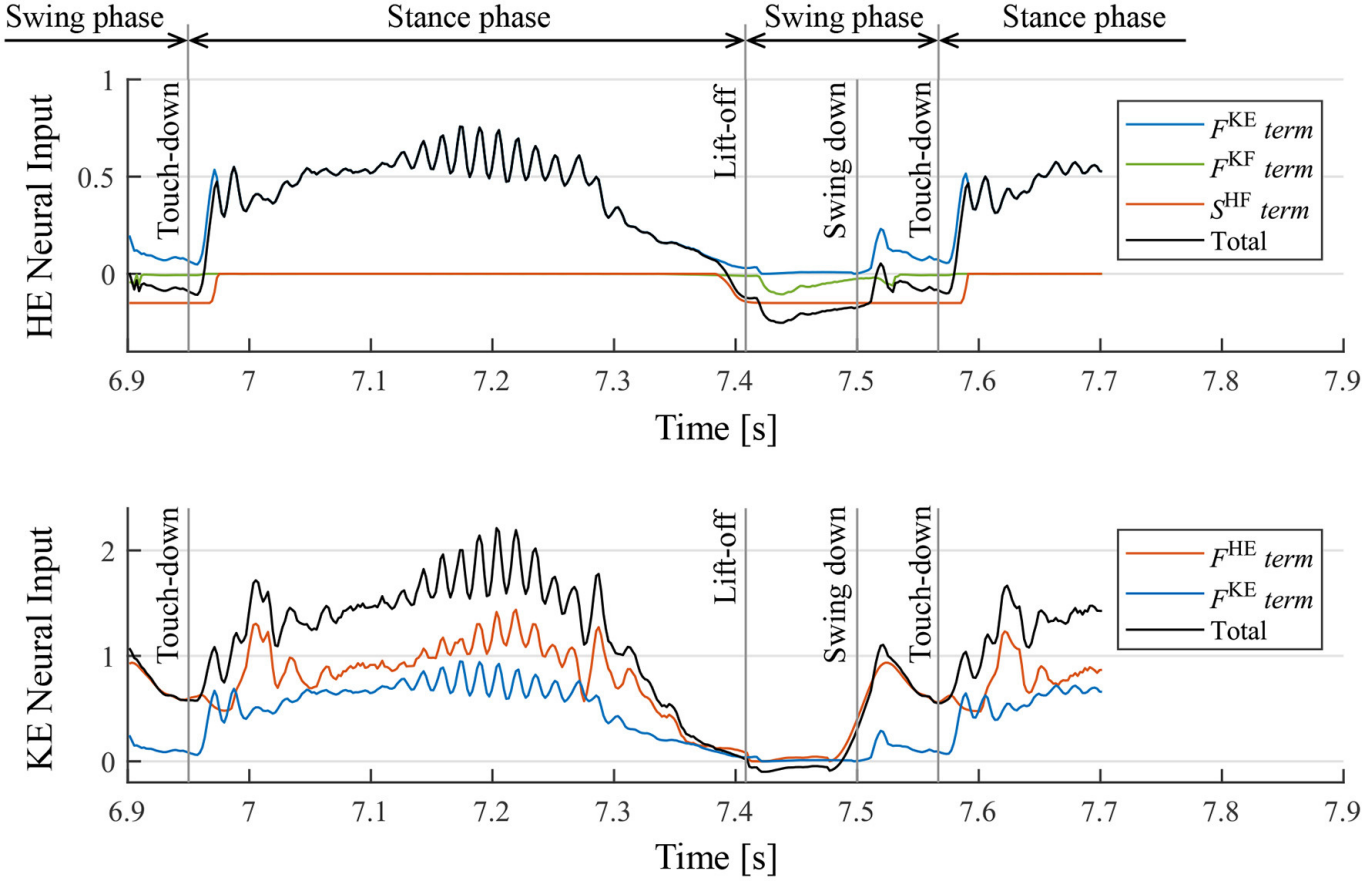
Neural input to interneurons of the hip extensor (HE) and knee–ankle extensor (KE) and the breakdowns. *F*^KE^, *F*^KF^, and *F*^HE^ are the magnitude of the muscle force feedback, and *S*^HF^ is the inhibitory neural input from the interneuron of the antagonist muscle. It should be noted that the sum of the knee–ankle extensor neural inputs includes an offset of –0.1.

Moreover, we investigated the mechanism of the motion generation phenomenon using the proposed reflex circuit. Figure 10 presents the values of each term on the right-hand side of Equation (9) for the hip and knee–ankle extensors, displaying the neural input from each feedback pathway and the total neural input to the interneurons^2^. In Figure 10, before the touch-down, the hip extensor is inactive, the knee–ankle extensor is activated, and the leg is located down the front. After the touch-down, tension is generated in the hip and knee–ankle extensors, and the terms of muscle force feedback, *F*^HE^ and *F*^KE^, became larger, resulting in the activation of both hip and knee–ankle extensors. At the time of the stance-to-swing transition, the terms of *F*^HE^ and *F*^KE^ become smaller, owing to the unloading of the weight on the leg; the hip and knee–ankle extensor muscles become inactive. At that time, the hip and knee–ankle flexors are activated, owing to the reduced inhibitory effect from the extensor interneurons, which produces a swing motion. At the timing of the leg swinging down, when the leg is moved forward, the term of *F*^HE^ on the knee–ankle extensor becomes larger. This implies that the hip extensor muscle is fully stretched, and it generates tension in the late swing phase. This activates the knee–ankle extensor muscle, which results in the swinging down of the leg. Subsequently, the swing-to-stance transition occurs at the timing of touch-down, and the walking motion is generated by repeating these sequences.

### 5.3. Walking Experiment With Different Initial States

In order to investigate the convergence property of the gait emerging from the reflex circuit, we conduct walking experiments from different initial conditions. When using the reflex parameters in Table 1, all the legs remain forward and stationary in the air. Therefore, to prepare the different initial conditions, we set the input bias $s_{0}^{\text{HE}}$ of the hip extensors to different values until just before the start of the walking experiment. The different bias values for $s_{0}^{\text{HE}}$ force the hip extensors to be activated or deactivated, as a result, we prepare two initial positions, one with the leg forward ($s_{0}^{\text{HE}}=-1$) and one with the leg backward ($s_{0}^{\text{HE}}=1$). By combining the two initial positions of each leg, we prepared the initial state of Table 2.

**Table 2:** List of initial conditions (value of $s_{0}^{\text{HE}}$) at the beginning of the walking experiment.

**Initial state**	**Values of** $s_{0}^{\text{HE}}$			
	**RF**	**LF**	**LH**	**RH**
A	−1	−1	−1	−1
B	1	−1	−1	−1
C	−1	1	−1	−1
D	1	1	−1	−1
E	−1	−1	1	−1
F	1	−1	1	−1
G	−1	1	1	−1
H	1	1	1	−1

Note that we fixed the initial state of the right hind leg to $s_{0}^{\text{HE}}=-1$ to evaluate the stability of the gait by the phase difference between each leg and the right hind leg.

The phase difference of the leg *k* ∈ {RF, LF, LH} to the right hind leg, Φ_*k*_, was calculated by the following equation based on Rosendo et al. (2014) using the ground contact time;

(11) $$\begin{array}{l} \Phi_{k}=2\pi \frac{T_{k\left(\text{actual}\right)}-T_{\text{RH}\left(\text{previous}\right)}}{T_{\text{RH}\left(\text{next}\right)}-T_{\text{RH}\left(\text{previous}\right)}}, \end{array}$$

where *T*_*k*(actual)_ is the contact time of each leg *k*, *T*_RH(previous)_ and *T*_RH(next)_ is the previous and next contact time of the right hind leg respectively. In Equation (11), since the phase difference between leg *k* and the right hind leg is calculated in the range of [0, 2π), the value of Φ_*k*_ changes drastically around 0 and 2π, e.g., it becomes 0 when exceeding 2π and 2π when falling below 0. Therefore, in order to prevent this drastic change in value, when the phase difference Φ_*k*_ converges around the 0 or 2π, we calculate the phase difference within the range of [−π, π) using the following equation;

(12) $$\begin{array}{l} \Phi_{0k}=\{\begin{array}{ll} \Phi_{k} & \Phi_{k}<\pi \\ \Phi_{k}-2\pi & \Phi_{k}\geq \pi \end{array}. \end{array}$$

**Figure 11:**
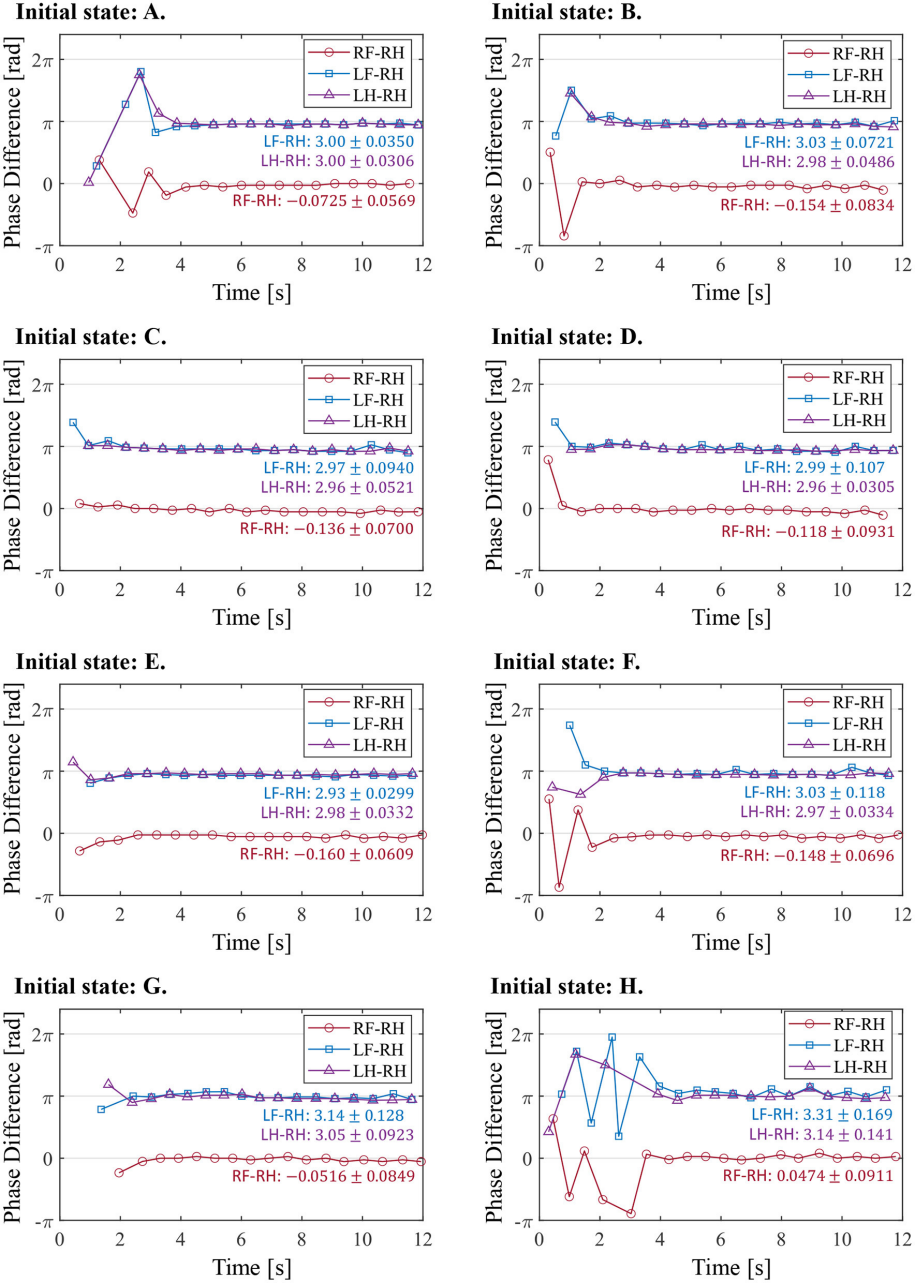
The phase difference between each leg and the right hind leg in the experiment from the different initial conditions. The initial states **(A–H)** correspond with those in Table 2. The figure also shows the mean and standard deviation of each phase difference after 4 s, when the gait was stabilized.

Figure 11 illustrates the experimental results from the initial state in Table 2. In the results from all initial conditions, the RF–RH phase difference Φ_RF_ converges near 0 rad, and the RF–RH phase difference Φ_LF_ and LH–RH phase difference Φ_LH_ converges near π rad. The results indicate that the front–hind legs are in the in-phase, and the left–right legs are in anti-phases, thus the robot produced a steady pace gait in all experiments.

### 5.4. Reproduction Experiment of Prolongation of Stance Phase via Nerve Stimulation

In the experiments by Whelan et al. (1995), electrical stimulation of the afferent nerves from the ankle extensor muscles prolonged the stance phase in walking cats. In this section, we conduct a robotic experiment using a similar condition as that in the cat experiment to evaluate the proposed reflex circuit.

**Figure 12:**
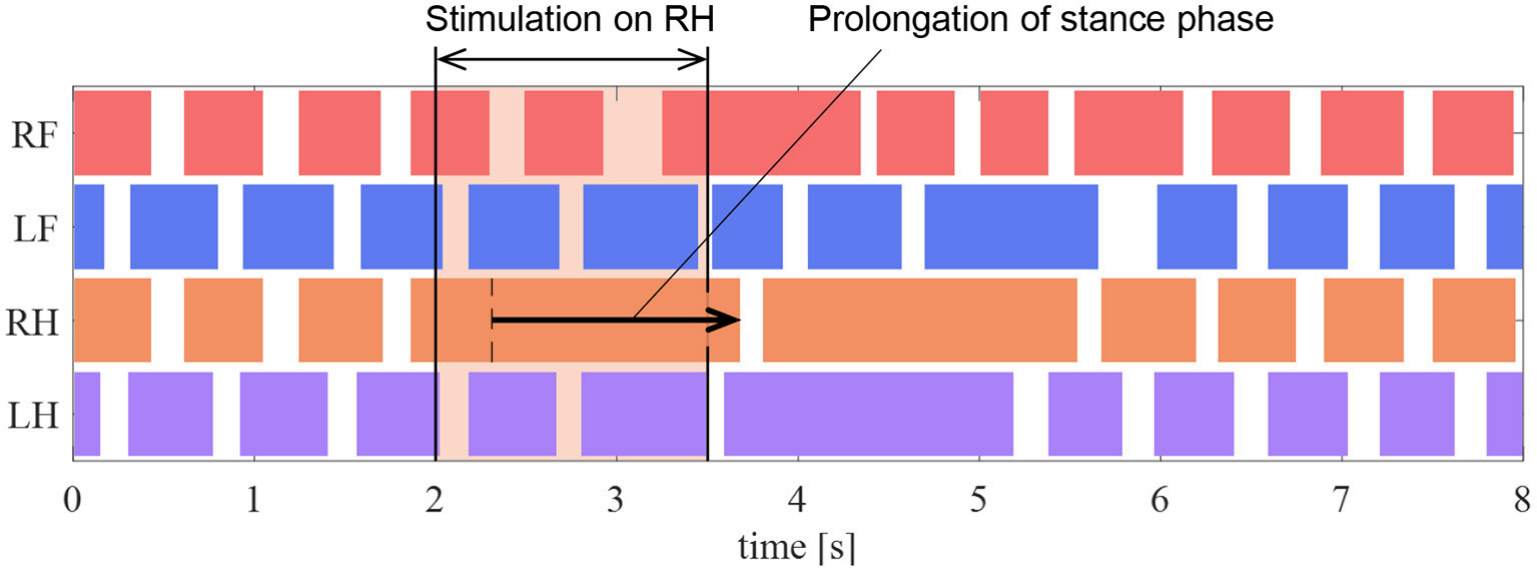
Gait chart in the stimulation experiment of the reflex pathway. The colored regions indicate ground contact.

In the experiment, the muscle force feedback pathway was stimulated after the quadruped robot produced a steady gait under the same conditions as those detailed in section 5.2. To reproduce the stimulation of afferent nerve which is considered to carry muscle force information of the ankle, the value of the afferent force feedback from the knee–ankle extensor was set as *F*^KE^ = 0.5 on the quadruped robot. The timing of the stimulation is 200 ms after the activation of the knee–ankle extensors as with the cat experiment. Figure 12 depicts the ground contact of each leg during the walking experiment. We stimulated the knee–ankle extensor afferent of the RH leg from 2 to 3.5 s. The contact time of the RH leg in Figure 12 during stimulation increased by 1.37 s compared with the normal gait. This indicates that the stance phase was prolonged. Thus, the proposed circuit with the reciprocal excitatory reflex reproduces the prolongation function of the stance phase observed in the walking cat experiments.

### 5.5. Experiment to Disable the Prolongation Function of the Stance Phase

In section 5.4, we demonstrated that the proposed circuit reproduced the prolongation function of the stance phase observed in the cat walking experiments by Whelan et al. (1995). This section investigates the effects of the proposed reflex pathway that provides the stance phase prolongation function for walking.

We investigated the effects of removing the reciprocal excitatory pathway in all the legs after the quadruped robot produced a steady gait under the same conditions as those in section 5.2. In the experiment, we set *F*^KE^ = 0 to disable the afferent feedbacks from the knee–ankle extensor to the hip and knee–ankle extensors when the angle of the hip joint was greater than 1.68 rad, thereby forcing the stance-to-swing transition. This corresponds to the temporary disconnection of the afferent nerve from the ankle extensor muscle in walking cats.

**Figure 13:**
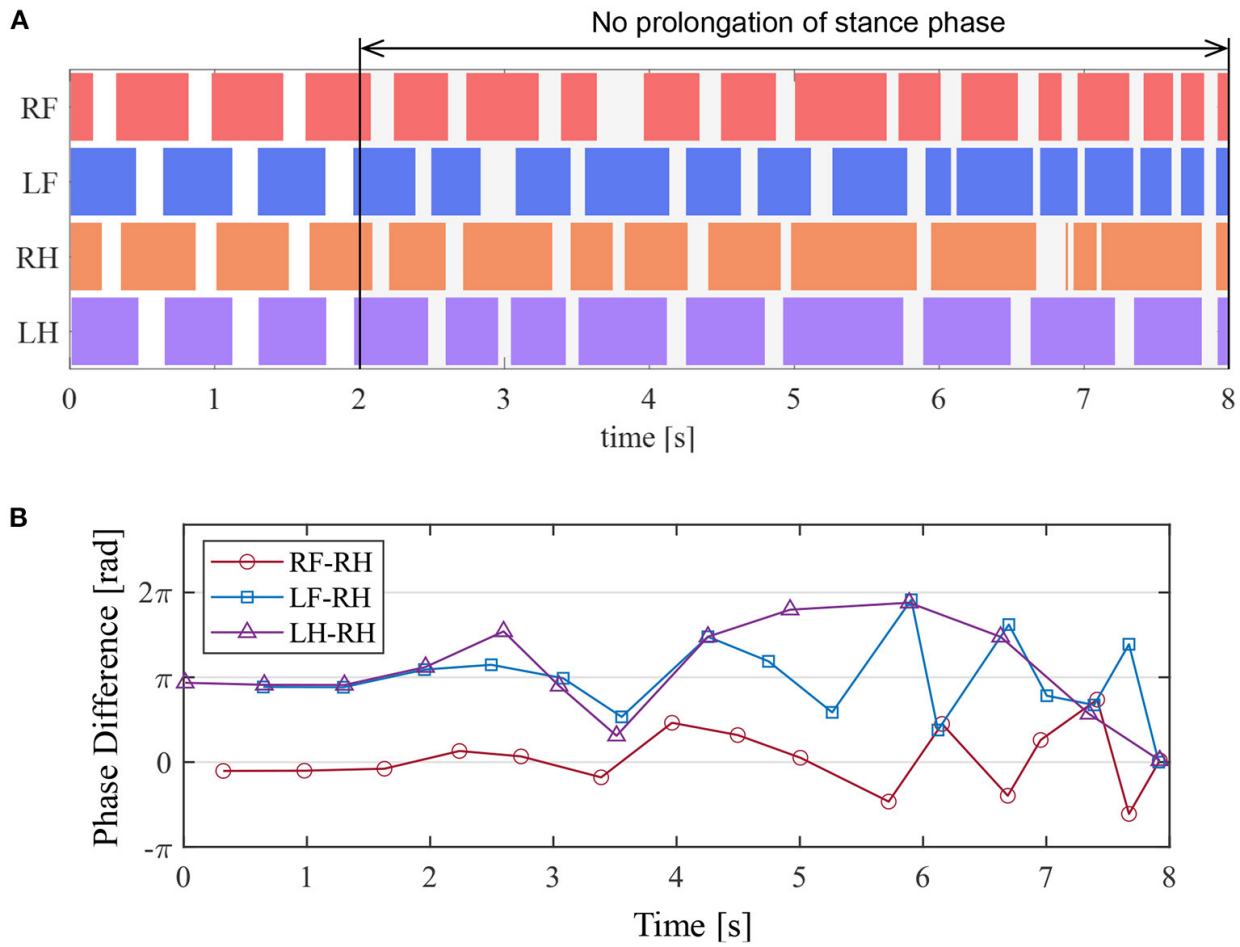
Experimental result to disable the prolongation function of the stance phase. **(A)** Gait chart. The colored regions indicate ground contact. **(B)** The phase difference between each leg and the right hind leg.

Figure 13A presents the ground contact for each leg during the walking experiment, and Figure 13B depicts the phase difference of each leg relative to the right hind leg. As shown in Figure 13, when the afferent feedback from the knee–ankle extensors was disabled after 2 s, the resulting gait was unsteady. This result indicates that the reciprocal excitatory reflex with a prolongation function of the stance phase stabilizes the gait pattern.

## 6. Discussion

### 6.1. Gait Emergence Mechanism With the Proposed Reflex Circuit

To reveal the reflex mechanism in animal walking, we proposed the simple reflex circuit based on walking cat experiments and evaluated it using the quadruped robot. In the walking experiment, the robot does not have CPGs or neural connections between its legs; however, the proposed reflex circuit exhibited rhythm generation and gait pattern adjustment, as shown in Figure 8. In this subsection, we discuss the mechanism of rhythm and gait emergence.

In the proposed reflex circuit, the rhythm generation function is considered a result of the self-excitatory force feedback of the knee–ankle extensor and the reciprocal inhibition of knee–ankle interneurons. These circuits activate the knee–ankle extensor to kick the ground when the reaction force is applied to the leg. Additionally, they activate the knee–ankle flexor to lift the leg when the leg is unloaded.

There are two possible reasons for the gait pattern adjustment. The first is the force–velocity characteristics of the muscle. Previous research by Masuda et al. (2017) has demonstrated that a quadruped robot without any closed loop controllers can lead to the emergence of some gaits depending on the applied voltage. This gait emergence phenomenon was caused by the characteristics of the DC motors that adjust the leg phases by slowing down the rotational speed when subject to the ground reaction force. The Hill-type muscle model used in this study also exhibits the force–velocity characteristics, which causes the contraction speed to slow down when a reaction force is applied. This is shown in Figure 3B and is similar to the DC motor's characteristics. Therefore, the contraction speed of the hip extensor muscles was adjusted by the muscle property during the support phase, in response to the ground reaction force, and it may have caused the gait emergence.

The second reason for the gait pattern adjustment is the prolongation function of the stance phase, owing to the muscle force feedback. Support legs continue to prolong the stance phase when the other legs are in the swing phase, owing to load concentration on the support legs. They shift to the swing phase immediately after the load is distributed when the other legs come into contact with the ground, thereby controlling the phase difference among legs. The studies (Ekeberg and Pearson, 2005; Maufroy et al., 2010; Rosendo et al., 2014; Habu et al., 2018) focusing the prolongation function also showed the similar phenomenon of gait stabilization.

### 6.2. Major Reflex Pathways Contributing to the Walking Motion Generation and Reproduction of Cat Behavior

This study presented a simple reflex circuit that generated leg trajectories and a steady gait and also reproduced the behavior of cats. This reflex circuit consists of reciprocal excitatory reflex between hip and knee–ankle extensors, self-excitatory reflex of knee–ankle extensors, and inhibitory reflex from knee–ankle flexors to hip extensors. In this section, we discuss that the reciprocal excitatory reflex between extensor muscles are particularly important in the generation of walking motions.

First, there are two major motor functions provided by the reciprocal excitatory reflex between extensors. The first is the knee extension associated with hip flexion at the end of the swing phase. When the hip extensor is fully stretched by the leg inertia at the end of the swing phase, the reflex from the hip extensor to the knee–ankle extensor is activated. As a result, the robot swing down the limb. The second is propulsion associated with leg loading during the stance phase. When an external force is applied to the knee–ankle extensor due to leg load in the early stage of the stance phase, the reflex from the knee–ankle extensor to the hip extensor is activated, resulting in a hip extension. Thanks to the reflex pathways, the hip extensor is activated according to ground reaction force on the knee–ankle extensor, as a result, the hip extensor produces forward propulsion when ground reaction forces and frictional forces increase.

Next, we explain how the reciprocal excitatory reflex between the extensor muscles affects the reproduction of cat behavior. The experiment by McVea et al. (2005) shows that assisting the flexor muscle movement during the swing phase accelerated the activation timing of the ankle extensor muscles. In the reciprocal excitatory reflex between extensor muscles we proposed, when the leg swing is accelerated by assisting the hip flexion, the activation of the knee–ankle extensors is advanced according to the hip flexion, thus reproducing the same phenomenon as in the walking cat. Moreover in an experiment of walking cats, Whelan et al. (1995) showed that electric stimulation on afferent nerves from ankle extensor muscles prolonged the stance phase. The same phenomenon was observed in the reciprocal excitatory reflex as shown in the section 5.4. Therefore, the reciprocal excitatory reflex between extensor muscles contribute to the generation of leg trajectories and the reproduction of behavior in cats, and may be a promising candidate for the structure of reflex pathways in animals.

### 6.3. Comparison With Previous Studies

As a closely related study to our result, a phase oscillator model of Maufroy et al. (2010) focusing on the prolongation of the stance phase in cats generated a steady gait in robot experiments.

On the contrary, in order to clarify the structure of the reflex circuit that generates the steady locomotion of cats, we implemented the reflex circuit model instead of a phase oscillator. reciprocal excitation we proposed agree with the previous models already know. In spite of the different implementations, since the result of this study is similar to those of previous studies, the reciprocal excitatory reflex between extensors we proposed has similar functions as the previous model of Maufroy et al. (2010).

In researches without using oscillator models or complex CPG models, a human model of Geyer and Herr (2010), cat model of Ekeberg and Pearson (2005), and cheetah model of Rosendo et al. (2014) produced leg trajectories and steady gait by the interaction between spinal reflexes, body dynamics, and environment. However, in these studies, the designers divided the walking motion into multiple phases (ex. stance, liftoff, swing, and touchdown phase) and designed a separate reflex rule for each phase. Therefore, it is not clear how these many reflex rules are integrated in the animal body, i.e., the overall structure of the reflex circuit that produces a steady gait and leg trajectory.

On the other hand, the major contribution of this study is clarifying the essential structure of the reflex circuit to produce a steady gait, which is the reciprocal excitatory reflex between hip and knee–ankle extensor muscles. This reciprocal excitatory reflex between extensors, which activates the hip extensor as the knee extensor is loaded, is a mechanically reasonable structure to produce forward propulsion when ground reaction force and frictional force increase. To the best of our knowledge, there are no existing examples of such a simple reflex circuit that generates the leg trajectory and steady gait autonomously. The proposed reflex circuit may be the current minimum sufficient structure for reflex circuits that reproduce animal gait.

In the simulation of a walking cat's hind legs by Ekeberg and Pearson (2005), the hip angle and the force of the ankle extensors were considered sensory candidates to initiate the stance-to-swing transition; a walking simulation was performed for these two candidates. As a result, the quadruped fell down in the case of the phase transition using the hip angle, and it walked with a steady gait in the case of the phase transition using the unloading of the ankle extensor. On the other hand, the experiment that disabled the prolongation function of the stance phase using the hip angle in section 5.5 demonstrated that the prolongation function based on the knee–ankle extensor force was more important for a steady gait than the phase transition based on the hip angle. Therefore, the results of this study support the hypothesis of Ekeberg and Pearson (2005) that ankle extensor unloading is the dominant factor in the stance-to-swing transition, instead of the hip angle.

### 6.4. Study Limitations

In this study, we showed that a reflex circuit with reciprocal excitation between extensor muscles produced leg trajectories and a steady gait, but the only gait observed within the parameters studied by the authors was the pace gait. It is not clear why the quadruped robot produced the pace gait, which is not a typical gait in cats. However, the models of Owaki and Ishiguro (2017) and Habu et al. (2018), which focused on the prolongation of the stance phase, produced multiple gait patterns such as walking, trotting, and galloping. Therefore, as in the previous studies, our model may also generate other gaits than pace by changing the body parameters, neuronal parameters, or adding reflex pathways. For example, the robot by Owaki et al. (2013) changed its gait from trot to pace by adding a mass to the robot and raising its center of gravity. Since our robot may also have a higher center of gravity than other robots in previous studies, we expect that our robot generates other gaits by changing the body parameters related to the center of gravity. In addition, there are 10 adjustable parameters in the reflex circuit. It is difficult to investigate the influence of all these parameters on the gait, so it is a future task to investigate it.

## 7. Conclusion

In this study, to clarify the structure of the reflex circuit that generates the steady locomotion of cats, we explored the reflex circuit using a quadruped robot platform that emulates the neuromuscular dynamics of animals. This circuit consists of a reciprocal excitatory muscle force feedback between the hip and knee–ankle extensors, a self-excitatory muscle force feedback in the knee–ankle extensors, and an inhibitory muscle force feedback from the knee–ankle flexor to the hip extensor, which is designed based on the results of walking cat experiments.

The major contribution of this study is clarifying the essential structure of the reflex circuit to produce a steady gait, which is the reciprocal excitatory reflex between hip and knee–ankle extensor muscles. In the walking experiments conducted on a quadruped robot with virtual muscles and a reflex circuit, a leg trajectory and gait pattern emerged, even though there were no CPGs or neural connections among the legs. Additionally, the robot reproduced the prolongation function of the stance phase using the stimulation of the ankle extensor nerves, which has been observed in walking cat experiments. Moreover, utilizing a robot with a reprogrammable reflex law in real time, we conducted an experiment to remove the reciprocal excitatory pathway between the extensors (the prolongation function of the stance phase) during walking. The absence of the reciprocal excitatory pathway reduced the gait stability of the robot. The results show that the reciprocal excitatory reflex between extensor muscles contribute to the generation of leg trajectories and the reproduction of behavior in cats. Therefore we believe that it may be a promising candidate for a key structure for generating the animal walking.

## Data Availability Statement

The original contributions presented in the study are included in the article/supplementary material, further inquiries can be directed to the corresponding author/s.

## Author Contributions

TT developed the robot, conducted the experiments and analysis, and wrote the draft of the manuscript. YM designed the study, contributed to the experiments and the interpretation of data, and assisted in preparing the manuscript. MI contributed to the interpretation of data and assisted in the preparation of the manuscript. All authors contributed to the article and approved the submitted version.

## Conflict of Interest

The authors declare that the research was conducted in the absence of any commercial or financial relationships that could be construed as a potential conflict of interest.

## Footnotes

^1^For the video of walking experiments, please refer to the following link: https://youtu.be/kxsAbZCh5KY.

^2^The neural input of flexor interneurons is not displayed in Figure 10 ; however, the flexor muscle becomes inactive when the extensor is activated for each joint. In contrast, the flexor is activated when the extensor is inactive, owing to the reciprocal inhibition of interneurons.
